# Integration of vibrotactile frequency information beyond the mechanoreceptor channel and somatotopy

**DOI:** 10.1038/s41598-017-02922-7

**Published:** 2017-06-05

**Authors:** Scinob Kuroki, Junji Watanabe, Shin’ya Nishida

**Affiliations:** NTT Communication Science Laboratories, NTT Corporation, Kanagawa, Japan

## Abstract

A wide variety of tactile sensations arise from the activation of several types of mechanoreceptor-afferent channels scattered all over the body, and their projections create a somatotopic map in the somatosensory cortex. Recent findings challenge the traditional view that tactile signals from different mechanoreceptor-channels/locations are independently processed in the brain, though the contribution of signal integration to perception remains obscure. Here we show that vibrotactile frequency perception is functionally enriched by signal integration across different mechanoreceptor channels and separate skin locations. When participants touched two sinusoidal vibrations of far-different frequency, which dominantly activated separate channels with the neighboring fingers or the different hand and judged the frequency of one vibration, the perceived frequency shifted toward the other (assimilation effect). Furthermore, when the participants judged the frequency of the pair as a whole, they consistently reported an intensity-based interpolation of the two vibrations (averaging effect). Both effects were similar in magnitude between the same and different hand conditions and significantly diminished by asynchronous presentation of the vibration pair. These findings indicate that human tactile processing is global and flexible in that it can estimate the ensemble property of a large-scale tactile event sensed by various receptors distributed over the body.

## Introduction

Touching an object produces a unique skin deformation reflecting mechanical interactions between the object and skin. The spatiotemporal features of skin deformation are neurally encoded in the activation patterns of multiple mechanoreceptor-afferent channels with distinct spatiotemporal tuning characteristics^1–5^, forming the basis of the content information (‘what’) of touch. The channels’ responses at each skin location are sent to the central nervous system (the contralateral side of the cortex) with the somatotopic organization preserved^6, 7^. The somatotopy forms the basis of the location information (‘where’) of touch.

Given this structure, the fundamental question in tactile processing is how the brain processes these multi-channel inputs from multiple skin locations to compute final touch perception. Specifically, we are interested in whether inputs from different channels and those from different skin locations contribute to the final perception in an independent manner or in an integrated one.

With regard to the inputs from different mechanoreceptor channels, a conventional view prefers independent processing, with each channel contributing to different aspects of the touch sensation^8–10^. Psychophysically, the detection sensitivity of each channel is not affected by adaptation or masking of the other channels^2, 11–13^. Physiologically, different tactile channels appear to be segregated from the periphery to, at least, the first stage of cortical processing, i.e., the primary somatosensory cortex (S1)^14–16^. However, while the psychophysical evidence for integration of multiple channels for vibrofrequency perception is still equivocal and not decisive^17–19^, recent physiological studies have shown that singles neurons receive peripheral inputs from multiple channels even in S1^20–22^.

Regarding interactions of peripheral signals from different skin locations, several studies have suggested interactions beyond strict somatotopic mapping. Physiologically, some neurons in S1 or higher areas have multi-finger/hand receptive fields, take projections from multiple peripheral neurons, and show inhibition/facilitation effects among them^23–29^. Psychophysical masking occurs even when similar input signals are presented to skin locations separated in the somatotopic map. While the masking effect generally decreases as a function of somatotopic separation, it remains even for bilateral inputs^30–32^. It is still unclear, however, whether this long-range interaction has some functional significance for tactile information processing or just reflects the brain’s practical limitation due to, for instance, insufficient attentional resources^33–35^. In summary, recent findings challenge the traditional view that neural signals from different mechanoreceptor channels are separately processed. It is also known that the tactile sensation of one body part is affected by stimulation of other parts. However, how cooperation between signals from different channels or locations contributes to tactile sensations remains poorly understood.

Here we psychophysically show that tactile inputs from different peripheral channels are integrated far beyond somatotopy, and lead to a better estimation of the tactile content information. In our first experiment, the participants estimated the perceived frequency of a target vibration while trying to ignore a distractor vibration, whose frequency was far different from the target and mainly activated a distinct channel^1–4^. The perceived frequency shifted toward the distractor’s frequency even though the vibrations were given to different fingers or even across hands. Moreover, we found in experiment 2 that when we asked the participants to estimate overall frequency of paired vibrations of different frequencies without specifying the target finger, they perceived an integrated signal with an intermediate frequency corresponding to the intensity-weighted average of the two vibrations presented to the different fingers/hands. Our results indicate that the brain can optimally integrate dissimilar vibration signals detected by different types of mechanoreceptors, despite their large separation in the somatotopic map, and effectively calculate ensemble property of inputs.

## Results

### Experiment 1: Frequency interaction across far different stimuli

**Figure 1:**
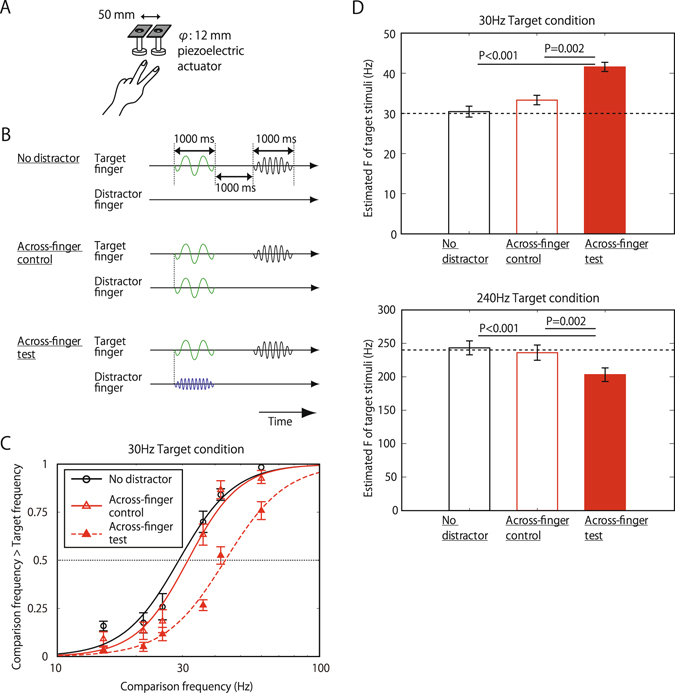
Frequency interaction Across-finger. (**A**) Schematic views of the set up. (**B**) Trial sequences for each distractor conditions, when the target frequency was 30 Hz. When the target frequency was 240 Hz, the green waves representing 30-Hz vibrations and a blue wave representing 240 Hz vibration were interchanged. Participants were asked to make binary response (higher, lower) about the perceived vibration frequency of the second/comparison vibration compared to the first/target vibration on the target finger, and they received a feedback beep after each response. The frequency of the target and distractor vibration was 30 Hz or 240 Hz, while that of comparison was varied. The perceived intensity of all stimuli were kept at the same level. As for the distractor vibration, the same-frequency vibration was presented in the control condition, while the other was presented in the test condition. The onset and duration of target and distractor stimuli were the same. Note that participants were explicitly told which fingers were the target and distractor. The target finger was fixed during a block and counterbalanced across blocks. (**C**) Psychometric functions representing the proportion of trials where participants judged the frequency of the comparison vibration to be higher than that of the target, which was fixed at 30 Hz. Each data point represents the average across ten participants. Error bars indicate ±1 SEM. (**D**) PSEs of the target vibration of 30 Hz (top) and 240 Hz (bottom) for each distractor condition. N = 10. Error bars indicate ±1 SEM.

If tactile signals from different channels and/or body locations were inseparably processed, the perception of an input to one channel/location on the body would be easily affected by the inputs to the other channels/body locations. In this experiment, we used a vibration frequency discrimination task to see how far different frequency/channel stimuli on far different locations interact with each other. Recently, Kahrimanovic *et al*.^36^ reported that when participants examined embossed dot surfaces on the target finger while ignoring the distractor surfaces on the neighboring finger, the roughness estimation of the target shifted toward that of the distractor texture (assimilation effect). Though their finding suggests an interaction of different signals across different body locations, the interaction could reflect low-level signal integration^23–29^. This is because the target and distractor stimuli were similar and likely to be encoded by common mechanoreceptor channels and because the body locations stimulated by the target and the distractor stimuli were close to each other — neighboring fingers. To see whether there are high-level interactions across different channels and locations, we presented 30- and 240-Hz vibrations as target and distractor stimuli, which were selected to effectively activate two distinctive vibrotactile channels: the RA (rapidly adapting) and PC (Pacinian) channels^1–4^. Each target and distractor were presented on the index or middle fingers of the left hand (Fig. 1A). Participants were asked to compare the perceived frequency of the first target vibration with that of the second comparison vibration presented to the target finger. The intensities of all stimuli were fixed at the same perceived intensities during the experiment. In the first condition, the target and distractor stimuli were presented on neighboring fingers (Across-finger-test condition). Participants were explicitly instructed to ignore the vibrations on the distractor finger and concentrate on the vibrations on the target finger (Fig. 1B). As control conditions, we conducted experiments with no distractor stimuli (No-distractor condition) or presented the same frequency stimuli as the distractor (Across-finger-control condition).

Figure 1C shows the averaged responses across ten participants for each condition with 30 Hz target. An increase in the comparison frequency smoothly raised the proportion of its being judged higher than the frequency of the target vibration. The psychometric function for the Across-finger-test condition (red filled triangles with red dashed line) horizontally shifted towards the higher frequency relative to the No-distractor (black open circles with black line) and the Across-finger-control condition (red open triangles with red line). The point of subjective equalities (PSEs), derived from the 50% point on the logistic function fitted to the psychometric function, were calculated for each participant for each condition and then averaged over the participants (Fig. 1D). Results showed an assimilation effect — the perceived frequency of the 30 Hz target vibration shifted toward a higher frequency when the frequency of the distractor vibration was 240 Hz (Across-finger test: 41.6 Hz on average) compared to when the distractor was absent (No distractor: 30.4 Hz; one-tailed paired t-test t = 4.75, p < 0.001) or when the frequency of the distractor was the same as that of the target (Across-finger control: 33.3 Hz; t = 3.89, p < 0.01). Similarly, a shift in perceived frequency of the target vibration toward the 30-Hz distractor was observed for the 240-Hz target stimuli (Across-finger test (203 Hz) vs No distractor (243 Hz); t = 2.03, p = 0.039; Across-finger test vs Across-finger control (236 Hz); t = 3.41, p < 0.01).

**Figure 2:**
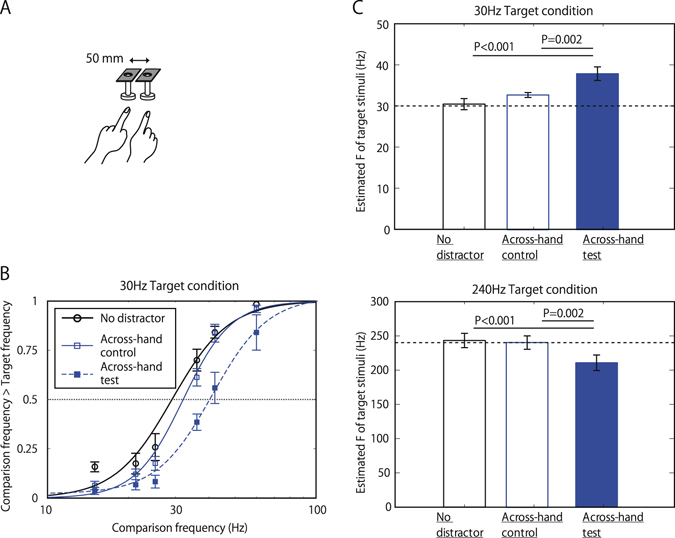
Frequency interaction Across-hand. (**A**) Schematic views of the set up. The distance between stimulators was the same to that in Across-finger conditions. (**B**) Psychometric functions of 30 Hz target condition. N = 10. Error bars indicate ±1 SEM. (**C**) Perceived frequency of the target vibration of 30 Hz (top) and 240 Hz (bottom). N = 10. Error bars indicate ±1 SEM.

For further consideration of the processing level of this interaction, we next tested whether the frequency interaction occurred across hands. As shown in Fig. 2A, we presented the distractor stimuli to the opposite hand (either to the index finger of the right hand or the middle finger of the left hand, not the same finger as the target) when the frequency of the distractor was different from that of the target (Across-hand-test condition) and when their frequency was the same (Across-hand-control condition). The perceived frequency of target stimuli for the Across-hand test condition was shifted toward distractor stimuli in comparison with the Across-hand control condition (37.9 Hz vs. 32.7 Hz for 30-Hz target, t = 3.07, p < 0.01; 211 Hz vs. 241 Hz for 240-Hz target, t = 2.81, p = 0.01) (Fig. 2B,C). This indicates that frequency interaction occurs even across further apart locations encoded by different hemispheres. Although the frequency shifts in Across-hand condition were slightly small in comparison to those observed in Across-finger condition, the difference was not statistically marginal (30 Hz target t = 1.57, p = 0.08; 240 Hz target t = 1.60, p = 0.07).

**Figure 3:**
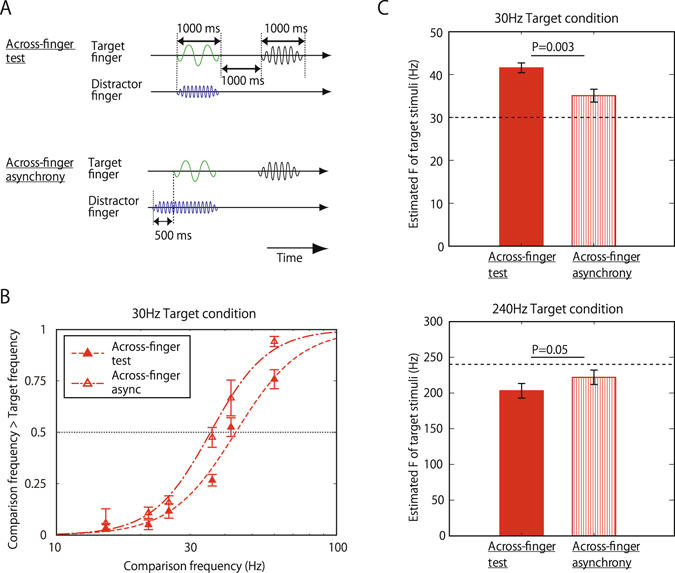
Effect of onset timing in frequency interaction Across-finger. (**A**) Trial sequences for each distractor condition, when the target frequency was 30 Hz. The onset of the distractor vibration was 500 ms earlier than that of the target vibration, and offsets of these two vibrations were the same. (**B**) Psychometric functions of 30-Hz target condition. N = 10. Error bars indicate ±1 SEM. (**C**) Perceived frequency of the target vibration of 30 Hz (top) and 240 Hz (bottom). N = 10. Error bars indicate ±1 SEM.

Finally, we changed the onset timing of the target and distractor stimuli to see the onset-synchrony effect (Across-finger-asynchronous condition), since it is known that onset synchrony is essential for signal integration in other modalities^37^. In this condition, as shown in Fig. 3A, the procedure was identical to that in the Across-finger-test condition except that the onset of the distractor stimulus was 500 ms (i.e., half the duration of the target stimuli) earlier than that of the target stimulus, while their offsets were the same. Here we found that the frequency interaction was significantly weakened for Across-finger asynchronous (35.1 Hz and 222 Hz) in comparison with Across-finger synchronous test (t = 3.67, p = 0.003 for 30 Hz target; t = 1.88, p = 0.048 for 240 Hz target) (Fig. 3B,C). This suggests that onset synchrony is a key factor for frequency interaction across different stimuli. When the onset is asynchronous between the target and the distractor, it becomes easier for participants to ignore the distractor, and accurately estimate the target frequency. The onset asynchrony however did not completely exclude the interaction at least in the present case. Compared with No-distractor condition, the perceived frequency of the 30 Hz target vibration was shifted toward a higher frequency (Across-finger asynchronous vs No distractor; t = 2.14, p = 0.04), while the difference was not statistically significant when compared to the case where the frequency of the distractor was the same as that of the target (Across-finger asynchronous vs Across-finger control; t = 0.72, p = 0.25). For 240 Hz target condition, the assimilation effect was not statistically significant in either case (t = 1.55, p = 0.08 for Across-finger asynchronous vs No distractor; t = 1.72, p = 0.06 for Across-finger asynchronous vs Across-finger control).

It is difficult to compare the absolute magnitude of the assimilation effect between the two target frequencies, since the shift was larger for 240 Hz on the linear scale, while larger for 30 Hz on the log scale. One may consider the log scale comparison to be more sensible for perceptual effects since frequency discrimination follows Weber’s law^38^, but the results of the next experiment support linear perceptual integration of vibration frequencies.

### Experiment 2: Frequency interpolation across far-different stimuli

The results of experiment 1 suggest that the activations of different types of mechanoreceptor afferents from different body locations interact with each other in an assimilative way. The compulsory intrusion of a non-target property to the perceptual judgment of the target might reflect a limitation on the brain’s capacity to accurately bind and segregate the content and the location information from multiple objects^33–35^. However, there may be some functional reasons why the brain shows this type of error. Assimilative interactions often result from neural integration of sensory signals. By signal integration, the brain attains abilities not only to increase the sensitivity of signal detection but also to estimate the ensemble property of the input signals as a whole^39–42^. In the present case, the brain may attempt to estimate the holistic and averaged properties of the tactile signals presented on different skin locations but likely caused by the same event since the signals are synchronized in time. As a result, an assimilation effect might have been observed in experiment 1. Although the observed effect size was much smaller than that expected from signal averaging, our participants were instructed to focus on one location and ignore the other to try to eliminate possible integration. Thus, in experiment 2, to better look at the mechanism of signal averaging across multiple inputs, we changed the procedure to the judgment of frequency of a pair of stimuli with a different frequency. As in experiment 1, we first report the results for single-hand conditions and then those for two-hand conditions.

In the Across-finger condition of experiment 2, 30 and 240-Hz vibrations were presented either to the index or the middle fingers of the left hand as a target “mixed” pair, followed by a pair of same-frequency vibrations whose frequency was varied across trials. Participants were asked to compare the perceived frequency of the target pair with that of a comparison pair presented to the same two fingers. In this experiment, the relative intensity of the two vibrations of target pair was varied. While the intensity of 30 Hz was fixed at the amplitude of 20 μm, the amplitude of the 240-Hz vibration was either 1, 2, or 4 μm. These conditions are named the 30 Hz-dominant, Equivalent, and 240 Hz-dominant conditions, respectively.

**Figure 4:**
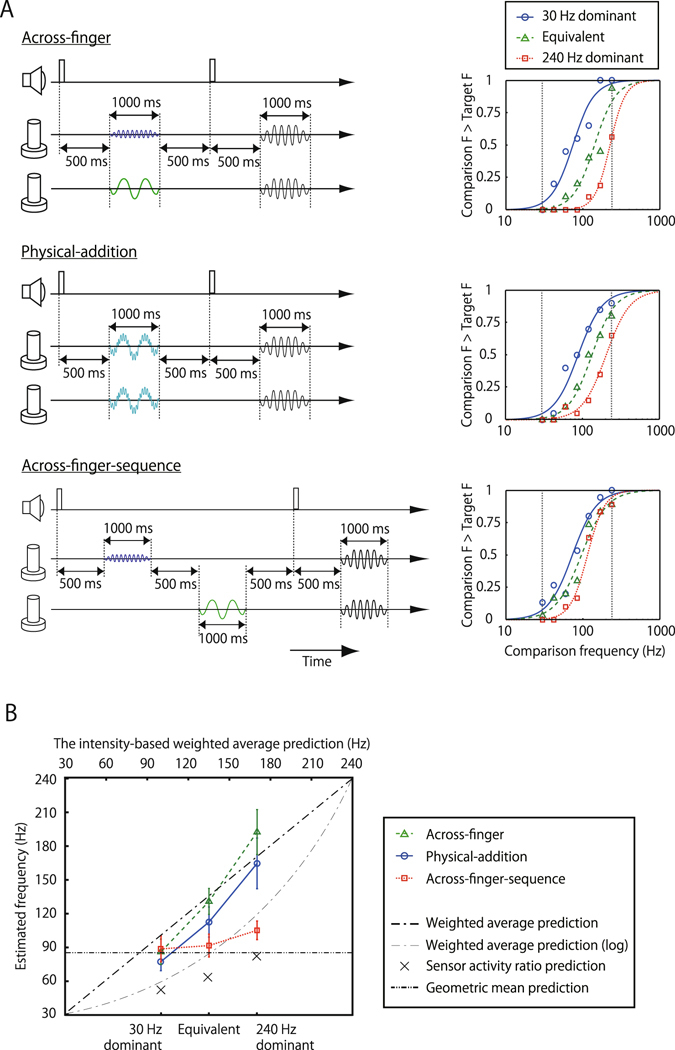
Frequency interpolation between low- and high-frequency vibrations. (**A**) Trial sequence and typical example of the individual data. The target pair and the comparison pair were sequentially presented on two fingers. Participants were asked to make binary responses (higher, lower) about the perceived vibration frequency of the second pair compared to the first. In this experiment, participants were not informed of any possible differences between paired vibrations, and they did not receive any feedback signal after their response. For the target pair, the amplitude of the 30-Hz vibration was fixed, while that of 240-Hz vibration was varied (0.5, 1, or 2 relative to 30- Hz intensity, each called 30 Hz dominant, Equivalent, 240 Hz dominant) to evaluate the perceived frequency shift. The order of target and comparison pair was randomized in Across-finger and Physical-addition conditions. The finger on which the 30- or 240-Hz vibration was presented as a test pair was randomized in Across-finger and Across-finger-sequence conditions. (**B**) Experimental results and theoretical values. Green triangles with a dashed line represent averaged PSEs of the target pair of the Across-finger condition from 11 participants, blue circles with solid line represent that of Physical-addition condition from 11 participants, and red square with dotted line represent that of Across-finger-sequence condition from nine participants. Data are plotted against the weighted average of 30- and 240-Hz vibrations. The dot-dash line represents intensity-based linear interpolation of 30 and 240 Hz, while the two-dot chain line represents the geometric mean of 30 and 240 Hz. The black cross represents the predicted frequency of the sensor activity ratio of the two channels (equivalent to arrows in Fig. 6A). Error bars indicate ±1 SEM. (see the detail in main text).

The top right panel of Fig. 4A shows the result for a typical participant for the Across-finger condition. An increase in the comparison frequency smoothly raised the proportion of its being judged higher than the frequency of the target/mixed pair. This suggests perceptual integration “averaging” of dissimilar vibrations across neighboring fingers, even though the two vibrations were not superimposed on the skin. What is more, the psychometric function for the 30 Hz-dominant condition (blue circles) horizontally shifted towards the lower frequency relative to the equivalent condition (green triangles), while that for the 240 Hz-dominant condition (red squares) shifted towards the higher frequency. The estimated PSE averaged over the participants (green triangles in Fig. 4B) indicated that the apparent frequency of the target pair was in the middle of the two original frequencies and was changed significantly by the intensity ratio (ANOVA F(2, 20) = 15.64, p < 0.001).

We presented simple mixtures of two frequency vibrations to the same fingers in a control condition, named the Physical-addition condition. A vibration signal having both 30- and 240-Hz components was simultaneously presented to both neighboring fingers. The trend obtained in the Physical-addition condition (the middle right panel of Fig. 4A) was quite similar to that obtained in the Across-finger condition. There was a significant change in the estimate of perceived frequency in accordance with the relative intensity ratios of the two vibrations (blue circles in Fig. 4B, F(2, 20) = 10.65, p < 0.0001).

One might suspect that the participant’s response in this experiment was not a perceptual judgment but a cognitive inference from the independently perceived frequencies of the two vibrations. To address this concern, we tested a third Across-finger-sequence condition (bottom left panel of Fig. 4A), in which low- and high-frequency vibrations were sequentially presented to the two fingers with a 500-ms interval. This interval was introduced to prevent perceptual integration of the two stimuli^36^. Participants were asked to estimate the averaged frequency of the two successive vibrations. Apart from the presentation timing, the procedure was identical to that in the Across-finger condition. The bottom right panel of Fig. 4B shows the result for a typical participant in the Across-finger-sequence condition. Though the estimated frequencies were midway between the two frequencies, they did not shift with the intensity ratio (red squares in Fig. 4B, F(2, 16) = 1.556, p > 0.24, ns). There was a significant interaction between the Across-finger and Across-finger-sequence conditions (F(2, 16) = 19.30, p < 0.001), which suggests distinct underlying mechanisms for each.

**Figure 5:**
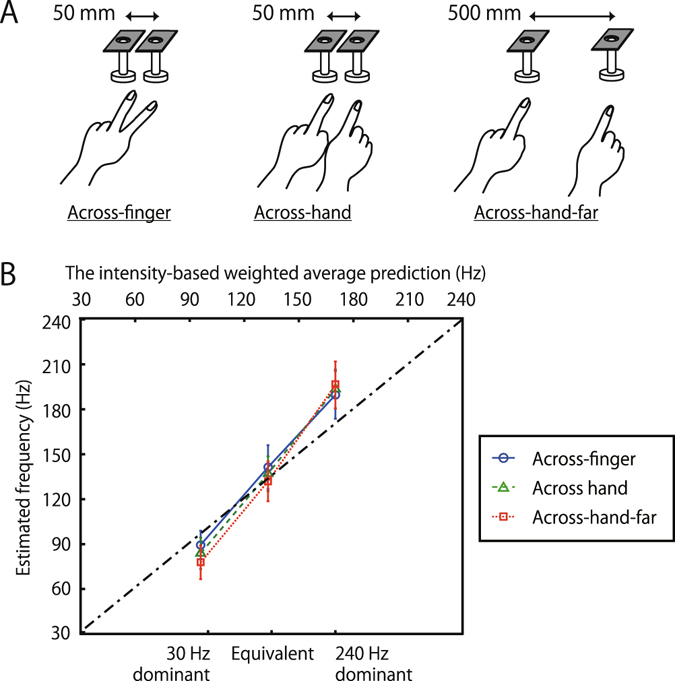
Effects of somatotopic and environmental separations in interpolation experiments. (**A**) Schematic views of the setup. (**B**) Experimental results. Blue circles with a solid line represent averaged PSEs of the target pair of the Across-finger condition, green triangles with dashed line represent that of the Across-hand condition, and red squares with a dotted line represent that of the Across-hand-far condition, all averaged across ten participants. The dot-dash-line represents intensity-based linear interpolation of 30 and 240 Hz. N = 10. Error bars indicate ±1 SEM.

To see whether this interpolation also occurs across hands, as we observed in experiment 1, we tested Across-hand conditions. The distance between the stimulators was 5 cm in the Across-hand condition as in Across-finger condition, while it was increased to 50 cm in the Across-hand-far condition (Fig. 5A). In the two Across-hand conditions, the stimulus somatotopic representation was the same, while spatiotopic representation was different. That is, the representations would be the same at the low-level areas in the brain but different at the higher areas, such as areas 5 and 7 in the posterior parietal cortex (PPC), where a tactile representation is combined with kinesthetic or visual information and remapped from the somatotopic (skin) coordinates to the environmental ones^43–45^. The results showed that the perceived frequencies in all conditions shifted according to the intensity ratio (Fig. 5B). Moreover, there was no significant difference between the Across-finger, Across-hand and Across-hand-far in PSE (F(2, 18) = 0.35, p = 0.7, ns) nor in JND (F(2, 18) = 0.47, p = 0.6, ns). We found that the frequency interpolation occurs even across hands, with no effect of inter-hand separation.

Our results suggest that low- and high-frequency vibrations activating different types of mechanoreceptors are perceptually integrated into a combined vibration whose frequency can be matched with an intermediate frequency. We found that the perceived frequency roughly agrees with the intensity-based interpolation, i.e., the weighted average of the two frequencies (diagonal line in Fig. 5B), with the weight determined by the relative intensity of each vibration. That is,

$${\rm{f}}=\frac{30\cdot {I}_{30}+240\cdot {I}_{240}}{{I}_{30}+{I}_{240}}$$

Here *I*_30_ and *I*_240_ denote perceived intensities of 30- and 240-Hz vibrations, respectively. This implies that the brain is able to estimate the frequency close to the optimal interpolation of the two signals as the ensemble property of the mixed pair. Note that the weighted average should be computed on the linear scale. The weighted average computed on the log scale (thin dot-and-dash curve in Fig. 5B) is much smaller than the perceived frequency. For instance, the predicted frequency is 135 Hz with linear scale and 85 Hz with log scale under the equivalent condition where the estimated PSEs of the 11 participants were 174, 173, 114, 110, 97, 110, 78, 153, 185, 99, 144 Hz.

It should be also noted that the PSEs for the Across-finger-sequence condition were close to the geometric mean (log non-weighted average) of 30 and 240 Hz (85 Hz, dashed line in Fig. 4B), as well as to the log weighted average. Since logarithmic scaling of vibro-frequency perception is suggested by nearly constant Weber fractions of frequency discrimination^38^, the participants’ responses in the Across-finger-sequence condition might be based on separate evaluations of the two vibration frequencies, followed by cognitive averaging of those estimations on a log frequency scale.

**Figure 6:**
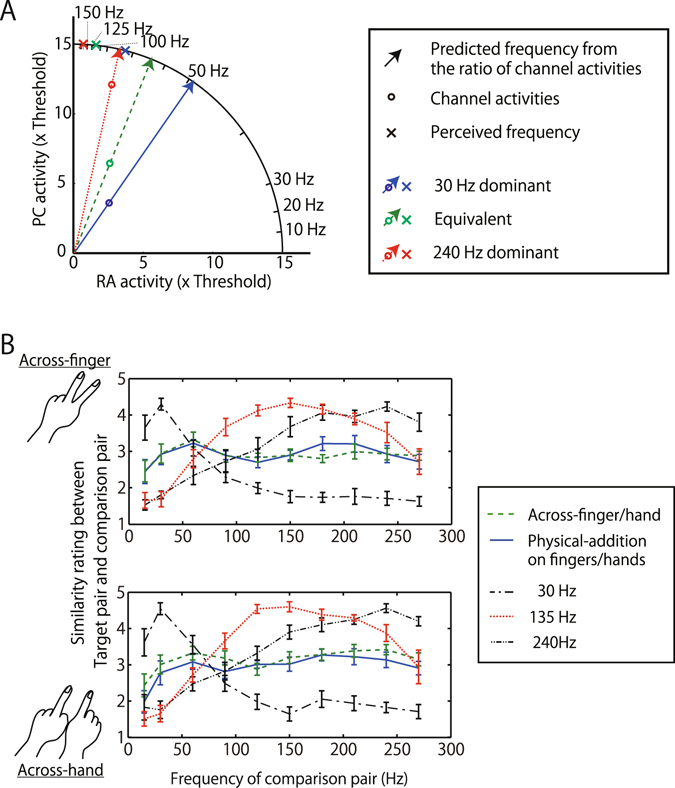
(**A**) Comparison between prediction of the ratio of channel activities and experimental results. Circles represent estimated channel activities for original stimuli in experiments based on sensitivity functions in previous research (1–5). Arrows represent predicted frequency from the ratio of RA and PC channel activities in the 30 Hz-dominant condition (blue arrow), Equivalent condition (green dashed arrow), and 240 Hz-dominant condition (red dotted arrow). These predicted frequencies are plotted as black crosses in Fig. 4B. Crosses represent matched/perceived frequencies in experiments, where each color represents each condition. (**B**) Similarity rating of the target pair and pair of sine-wave vibrations. Participants rated the similarity from 1 (dissimilar, easily-distinguishable) to 5 (very similar, indistinguishable). Note that 135 Hz (Red dotted line) is the weighted average of 30- and 240-Hz vibrations, though the rating distribution of this condition is far different from that of the target pair of Across-finger/hand or Physical-addition. N = 8. Error bars indicate ±1 SEM.

One may consider that frequency interpolation, or averaging, from two stimuli with different frequencies is conceptually similar to visual color mixing^46^. However, the computational method seems to be different. Since color perception is determined by the relative activities of three color channels, we calculated the simulated activity ratio of the RA and PC channels based on the reported sensitivity functions^1, 9^. The predicted frequency based on sensor activity ratio is represented as the black crosses in Fig. 4B and arrows in Fig. 6A. The pattern was very different.

The finding that the method of computing the tactile frequency from a mixed pair is different from that of color mixing is consistent with our finding from experiment 1. Since the observers retained some ability to separate the vibration component presented to one location from that presented to the other, the perceptually mixed frequency cannot be called a perfect metamer (perceptually equivalent stimulus) of a single sine-wave vibration with the matched frequency. It seems that the mixed stimuli and matched stimuli are the same in the frequency comparison domain but not necessarily the same in perceptual representation. To further consider the perceptual representation of the mixed-frequency stimuli, we estimated the subjective frequency profile using a frequency similarity task. For single-frequency vibrations (30, 135 and 240 Hz) and a mixed pair (30 + 240 Hz) presented with physical-addition or across finger(s) or hand(s), we asked the observers to judge the subjective similarity to a vibration with a single frequency selected from a wide frequency range. The results show that the perceived frequency tuning for the single-frequency vibrations is unimodal and has peaks at around the physical frequency (Fig. 6B). On the other hand, the perceived frequency tuning for the mixed pair is broad and has no clear peaks at around the component frequencies, regardless of the presentation condition. The results support our argument that the vibration frequency mixture is computationally different from a color mixture, in that it does not produce the perceptual metamer of a single-frequency vibration. The results also suggest that the subjective frequency profile is very broad and very similar regardless of whether the two vibrations are presented on the same finger, across fingers, or across hands.

## Discussion

The tactile system senses a variety of surface properties from vibrations given to the skin. Past studies have shown that low- and high-frequency vibrations, which are respectively associated with rough/coarse and smooth/fine textures, are encoded by distinct mechanoreceptor-afferent channels^1–4^. At the same time, signals from different skin locations are somatotopically projected to different loci in the primary somatosensory cortex^1–19^, but see refs 20–22. Although some interactions across channels or locations are known^30–32^, little is known about how the vibration signals are integrated across different channels and locations, and how the different signals produce the final vibration perception. Here we show that different frequency-channel stimuli interact with each other even when the two stimuli are presented on different fingers of different hands. First, when the participants were asked to judge the vibration frequency on one finger, they could not ignore the assimilation effect due to the other stimulus presented synchronously on a different finger. Second, when they were asked to judge the whole frequency of a pair of different-frequency stimuli, their answer was the result of intensity-based interpolation.

Consider first the integration of tactile signals across different skin locations. Some interactions between separate inputs have been reported at the neural and behavioral level. The receptive field of a somatosensory neuron, within which input signals are integrated, tends to grow large as the processing goes from lower to higher cortical areas. For example, typical receptive fields span a single finger, multiple fingers, and bilateral areas in area 3b, 1, and 2 in S1, respectively^27, 47–49^. Detection of a vibration signal is impaired (masked) by another vibration of similar frequency (masker) even when the masker is presented at a separate location on the body surface, although the masking magnitude tends to decrease as the separation increases. These findings indicate tactile signal interactions are not limited to proximal inputs, but the functional meaning of the interactions was not clear. Our findings in experiment 1, though similar to those previously reported by Kahrimanovic *et al*.^36^, clarified that the assimilation effect should be ascribed to signal integration in central processing, not in low-level neural interaction. This is because the two signals to be integrated are widely separated in somatotopic space, as well as in receptor space. In addition, the integration process is not hardwired, since it became weak when the input signals are asynchronous in time.

The somatotopic map can be regarded as the mechanism to correctly bind the content “what” and the location “where” of tactile signals. Several misbindings of “what” and “where” signals are known, and they can be ascribed to misaddresses in processing of the somatotopic map. For instance, patients with body amputations sometimes claim phantom pain from the absent body part^50, 51^. This could be due to reorganization of somatotopic representation. Participants often report a reversal in the subjective touch order when their left and right hands are sequentially touched while their hands are crossed^52, 53^. This effect can be ascribed to errors in remapping the perceptual representation from the somatotopic space to the external space. One may consider the non-somatotopic integration we found as another example of the misbinding of “what” and “where” signals. However, we think that our non-somatotopic integration does not reflect an error, but an elegant binding strategy of the tactile system. Signal coincidence in space and that in time are two major cues of signal binding, with the weight being dependent on the modality. There is no doubt that spatial (somatotopic) coincidence is a strong cue for tactile binding, as well reflected in the masking effect^30–32^, but the present finding suggests that temporal coincidence (synchrony) is also a strong enough cue to overcome the lack of spatial coincidence. The tactile signal processing is more global and more flexible than previously thought.

Consider next the integration of tactile signals across different types of mechanoreceptors. Previous studies have highlighted the divisions of roles played by each channel. For neural representations, it has been assumed that the different channels are segregated at least through the first stage of cortical processing S1^14–16^. For perception, the contribution of each channel has been individually considered: the slowly adapting I (SAI) channel dominantly responds to input pressure and might contribute to shape and motion perception; the SAII channel responds to skin stretch; the rapidly adapting (RA) channel responds to flutter and contributes to coarse texture perception; and the Pacinian (PC) channel responds to vibration and contributes to fine texture perception [see review for ref. 54]. Recent physiological studies, however, showed the possibility of information integration across different channels at a very early level of the somatosensory cortex^20–22^. To test the possible contribution of the combined activity of different channels to vibrotactile frequency perception, a few psychophysical studies have examined whether a change in vibration amplitude could shift the perceived frequency in the expected direction^18, 19^ or whether there was a labeled-line structure expected from the cross-channel encoding of frequency^17^. However, the results of these studies were equivocal and not decisive. Our interpolation illusion provides the first clear behavioral evidence for the contribution of cross-channel information to tactile processing, at least, in the estimation of vibrotactile frequency. We hypothesize that the cross-channel interaction in frequency perception takes place in a hierarchical way. That is, at first the frequency and intensity of each frequency are analyzed within each channel, and then the outputs from different channels are combined at a higher processing stage in an optimal fashion. We suspect that this tactile processing is more similar to a mid-level visual processing that gives rise to crowding and ensemble coding^39–42^ than to an early visual processing yielding color mixing.

Concerning neural locus of frequency interpolation, recent studies have revealed the convergence of RA and PC signals to neurons in the early somatosensory cortex (areas 3b, 1), and it seems that the two channels are represented in different manners (spike rate for RA and spike timing for PC) and thus still functionally segregated at this stage^20–22^. Rather, our results suggest that the non-somatotopic cross-channel interaction of “frequency” information occurs at relatively higher stages of tactile processing (at least higher than areas 3b and 1), since the frequency interpolation must take place after the convergence of the tactile signals from different hands, which is mainly found in area 2 in S1^27, 47^. On the other hand, our failure to find an effect of bilateral hand separation suggest that it might take place before the tactile representation from the somatotopic (skin) coordinates is remapped to the environmental coordinates, which is observed in such areas as PPC^43, 45^. It should be noted, however, that Rahman and Yau (personal communication) recently reported that an across-hand vibrofrequency assimilation effect similar to ours in experiment 1 (though using only PC dominant vibrofrequencies and different finger conditions) is reduced as a function of bilateral hand separation. The effect of body posture on tactile signal integration should be further investigated in the future.

To demonstrate non-somatotopic integration of tactile signals, we presented a pair of vibrations to different hands. A remaining question is whether the fingers or hands have a special status in non-somatotopic integration, or whether similar signal integration generally occurs between any two sites on the body regardless of the distance separating them. In relation to this point, we are interested in a recent report that shows that the perceived frequency of a tactile vibration shifts toward the frequency of a simultaneously presented auditory sound^55^. It is possible to consider this intriguing phenomenon as a cross-modal version of the assimilation effect we found in experiment 1. If there are some mechanical overlaps between the two assimilation effects, it is unlikely that non-somatotopic tactile integration is body-specific. This is an important topic for future study.

In summary, we presented evidence showing that the perception of frequency/texture arises not only from neural firing for a specific mechanoreceptor channel at a specific skin location but also from the integration of synchronous inputs across different channels and different skin locations. The brain can estimate the “what” information of one external object/event by optimally integrating synchronous inputs despite a difference in the “where” information. This is a reasonable strategy, considering that we often touch and explore the outside world using multiple digits and both hands.

## Methods

### Participants

Twenty-six (seven males) naïve participants with normal tactile sensitivity (by self-reports), aged from 23 to 43 years participated the experiments. Although they varied in age, we did not find significant influence of age on our experimental results. All gave written informed consent before the start of the experiment. Recruitment of participants and experimental procedures were approved by the NTT Communication Science Laboratory Research Ethics Committee, and were conducted in accordance with the Declaration of Helsinki.

### Apparatus

Tactile stimuli were generated by a stack-type piezoelectric actuator (NEC TOKIN, Sendai, Japan, ASB680). Two stimulators were used to present vibrations to the two fingers; each can be independently driven to normally deform the skin with a maximum of 800 N force. Stimulators were vibrated by a position control method so that they could accurately produce commanded displacement with a tolerance of few nanometers. The diameters of stimulators were 12.0 mm, and the edges of the stimulators were separated from the rigid surround of the metal boards by a 1.0-mm gap [resembles Verrillo’s work^56^]. The rigid surround limits the spread of surface waves of the skin^13^. The stimulators always contacted the fingers throughout the experiment, and the distance between them was kept constant at 5 cm, except in Across-hand-far condition in experiment 2, where it was kept at 50 cm.

### Procedure

Experiment 1. Ten participants participated. The experimental setup is shown in Fig. 1A. A participant sat at a table and placed the index and middle fingers of the left hand on stimulators through holes in a metal board. In Across-hand conditions, he/she placed the index finger of the right hand and the middle finger of the left hand on the stimulators. A two-alternative forced-choice method was used to determine the frequency discrimination performance. One finger was used for the task; the other was for distraction. First a 1000-ms target vibration was presented to the target finger, followed by a 1000-ms comparison vibration, separated by an interval of 1000 ms. The frequency of the target vibration was either 30 or 240 Hz, while that of the comparison vibration was one of six frequencies, which were 0.5, 0.71, 0.84, 1.2, 1.4, or 2.0 times the target frequency. The intensity of the 30-Hz vibration was fixed at the amplitude of 20 μm (corresponds to around five times the threshold with this stimuli). That of the 240-Hz vibration was fixed at 2 μm, which was matched in the perceptual intensity based on the results of a preliminary experiment. Also, the amplitudes of comparison vibrations were chosen individually for each frequency so that they matched with that of a 20-μm 30-Hz sine vibration. The preliminary experiment was done with individual participant, and the amplitudes used by one exampled participant was 30, 24, 22, 20, 13, 10, 5, 3, 2, 2, 3, 3 μm for 15, 21, 25, 36, 42, 60, 120, 170, 202, 288, 336, 480 Hz. The onset and offset of each vibration was modulated with a raised cosine window with a frequency of 20 Hz to reduce the saliency of the rise and decay of skin deformation. Except for the No-distractor condition, a distractor was presented on the other finger. A 1000-ms distractor vibration was synchronously presented on the distractor finger with the target vibration (the first vibration on the target finger) in all conditions except the asynchronous condition, where a 1500-ms distractor stimulus was presented 500 ms prior to the target stimulus so that the onset was asynchronous while the offset was synchronous. The frequency of the distractor was either 30 or 240 Hz: a different frequency from the target vibration was chosen for test conditions, while the same frequency was chosen for control conditions. Note that at the beginning of each block, participants were told which finger would be the target, which was fixed during a block, and instructed to ignore any stimuli presented on the other (distractor) finger. Participants made binary responses as to which of the two vibrations (the first or the second vibration on the target finger) had the higher frequency using a foot pedal. After each response, feedback information was given by means of an auditory signal to let the participant know which percepts corresponded to higher and lower vibrations. We expected that this procedure would maximize the resulting discrimination performance. They performed experiments with their eyes open to maintain their arousal level, but they could not see the vibration of the actuator, and they wore earplugs and headphones. White noise was presented continuously throughout the experiment from the headphones to mask any subtle sound made by the tactile stimulation. There were two target frequencies (30 and 240 Hz), six distractor conditions (No distractor, Across-finger-control, Across-finger-test, Across-hand-control, Across-hand-test, and Across-finger-asynchronous), and the number of trials for each combination was 120 (20 trials for each comparison frequency). The No-distractor and Across-finger conditions were tested in the same session, and sessions were divided into ten blocks. Across-hand conditions were tested in the same session, and sessions were divided into five blocks. Within each block, the distractor condition and frequency of comparison vibrations were randomized and the target frequency and target finger were fixed.

Experiment 2. Eleven participants participated for the Across-finger condition, eleven for the Physical-addition condition, and nine for the Across-finger-sequence condition. Participant groups were different between the Across-finger condition and Physical-addition condition, while the group for the Across-finger-sequence condition was part of that for the Across-finger condition. The experimental setup and procedure were nearly identical to that of experiment 1, but mainly differed in the following aspects: two pairs of vibrations were presented and the perceived frequency was compared between pairs; participants were not informed about a possible discrepancy in frequency between the paired vibrations; and no feedback que was presented for the task.

The experimental procedure is shown in Fig. 4A. A trial started with a beep sound. After an interval of 500 ms, the first pair of vibration stimuli was delivered to two fingers, followed by the second pair with an interval of 1000 ms, including a second beep at an intermediate between the two pairs. Participants made binary responses as to whether the second pair of stimuli was higher or lower in frequency than first pair by clicking a mouse with their right hand. One stimulus of the pair was a target; the other was a comparison (sine-wave vibration). The waveform of the target pair differed among conditions. For the Across-finger/hand conditions, a 30-Hz vibration and 240-Hz vibration were simultaneously presented on each finger as the target pair. Which finger was given which frequency was randomly changed across trials. For the Physical-addition condition, the target pair was the addition of a 240-Hz vibration to a 30-Hz vibration, presented on both fingers simultaneously. For the Across-finger-sequence conditions, a 30-Hz vibration and 240-Hz vibration were presented sequentially with a 500-ms interval. For all conditions, the amplitude of 30-Hz vibrations was fixed at 20 μm; that of 240-Hz vibrations was selected from 1, 2, or 4 μm (around half, the same, or double the intensity of 30-Hz vibrations). The frequency of the comparison pair was chosen from seven frequencies between 30 to 240 Hz, whose perceptual intensity was matched with that of a 20-μm 30-Hz sine wave. There were three intensities of 240 Hz (1, 2, 4 μm), three target conditions (Across-finger, Physical-addition, Across-finger-sequence), and the number of trials for each combination was 140 (20 trials for each comparison frequency). Each target condition was divided into ten blocks. Within each block, the order of the target and comparison pair was randomized between trials except in the Across-finger-sequence condition. The intensity of 240 Hz in the target pair and the frequency of the comparison stimulus were randomized.

Experiment 3. Ten participants participated. The experimental setup and procedure were identical to those of the Across-finger condition in experiment 2 except for the distance between the stimulators, which was kept at 50 cm for the Across-hand-far condition and at 5 cm for the rest (Fig. 5A). There were three intensities of 240 Hz (1, 2, 4 μm), three target conditions (Across-finger, Across-hand, Across-hand-far), and the number of trials for each combination was 140 (20 trials for each comparison frequency). Each target condition was divided into ten blocks.

Experiment 4. Eight participants participated. A rating method was used to determine the similarity between the target pair and comparison pair. The stimulus sequence was nearly identical to that for the Across-finger and Physical-addition conditions in experiment 2. Participants rated the similarity between the target pair and the comparison pair, from 1 (dissimilar, easily-distinguishable) to 5 (very similar, indistinguishable). Five different types of the target pair were tested for the Across-finger and Across-hand posture: target pair of Across-finger condition, target pair of Physical-addition condition, a pair of 30-Hz vibrations, a pair of 135-Hz vibrations, and a pair of 240-Hz vibrations. Note that 135 Hz is the weighted average of 30- and 240-Hz vibrations. The intensity of the 30-Hz vibration was 20 μm and that of 240 Hz vibration was 2 μm. The frequency of the comparison pair was 10, 30, 60, 90, 120, 150, 180, 210, 240, or 270 Hz. The number of trials for each combination was 200 (20 trials for each comparison frequency).
